# One More Word

**Published:** 1895-12

**Authors:** 


### ONE MORE WORD.

If the writer of the protestation to the Association of Faculties did not wish to crush out this movement, why did he not write a direct letter to the organization's officers, or delegate a representative to attend its sessions and present there his views? Why did he prepare some two or three hundred copies of a letter and ask for their general distribution among many who, not like himself, have little conception of the difficulties that for so many years surrounded its achievement? Why, if so earnestly interested in its real success, were such demands made for its dissolution at an hour when it needed the strong arms of support of every true friend of higher veterinary education, when every veterinary college of North America was exhibiting the keenest interest in the success of this movement? Why should one who for so many years was an acknowledged leader attempt to tear down that for which ten years of the most exacting labor and sacrifices had been made, at the hour of its highest fruition and greatest opportunities will ever remain a source of surprise and regret. Why should he wish to clothe such an organization with legal fetters, when he acknowledges no allegiance to the country of his adoption, and therefore could not be bound by these? Science may know no country; scientists may care little for citizenship in their chosen fields of labor; but let it not be forgotten that American citizenship and all its sacred privileges are dear to the hearts of every American-born child, and when scientists wish to be without country they should be consistent, and not ask for conditions to which they would not be amenable. They should confine their obligations to the safety of those moral pledges which bind men together for the common good and advancement of the cause they represent. Yea, more; they should not adopt names for institutions, publications, hospitals, etc., which are misleading and, to say the least, not in good taste nor excusable on the ground of being wholly scientific.

